# Temporal Immune and Metabolic Shifts Drive the Anti‐Tumor Efficacy of Resiquimod‐Loaded Nanoparticles in Peritoneal Carcinomatosis

**DOI:** 10.1002/adhm.71424

**Published:** 2026-07-08

**Authors:** Vanessa Chan, Po‐Han Chao, XuXin Sun, Jun Han, Sarah MacPherson, Lucas J. Andrew, Noah Y. Brittain, Tien Do, Marcel B. Bally, Julian J. Lum, Shyh‐Dar Li

**Affiliations:** ^1^ Faculty of Pharmaceutical Sciences University of British Columbia Vancouver British Columbia Canada; ^2^ Basic and Translational Research Department BC Cancer Research Institute Vancouver British Columbia Canada; ^3^ Faculty of Medicine University of British Columbia Vancouver British Columbia Canada; ^4^ Division of Medical Sciences Genome British Columbia Proteomics Centre University of Victoria Victoria British Columbia Canada; ^5^ Trev and Joyce Deeley Research Centre BC Cancer Victoria British Columbia Canada; ^6^ Department of Biochemistry and Microbiology University of Victoria Victoria British Columbia Canada; ^7^ Faculty of Chemistry University of British Columbia Vancouver British Columbia Canada

**Keywords:** cancer immunotherapy, colorectal cancer, metabolomics, nanomedicine, ovarian cancer, peritoneal carcinomatosis, tumor immune microenvironment

## Abstract

Peritoneal carcinomatosis (PC) is an aggressive manifestation of advanced gynecological and gastrointestinal malignancies with high recurrence rates despite cytoreductive surgery and chemotherapy. We developed a cationic liposomal nanoparticle (DSTAP, ∼159 nm) to deliver and retain the toll‐like receptor 7/8 agonist Resiquimod (R848) within the peritoneal cavity, aiming to modulate the tumor immune microenvironment (TIME) and improve therapeutic efficacy. DSTAP‐R848 was evaluated in murine colorectal and ovarian PC models, alone or combined with oxaliplatin (Oxa). Survival, cure rates, and durable immunity following tumor rechallenge were assessed. Immune polarization was examined by incubating ascites and peritoneal fluid with naïve splenocytes, while flow cytometry and metabolomic profiling characterized intratumoral immune populations and metabolic changes. Combination therapy achieved cure rates of 80% in the colorectal model and 30% in the ovarian model, with no recurrence after rechallenge; Oxa monotherapy yielded no cures. Oxa+DSTAP‐R848 increased CD8^+^ T cells and M1 macrophages, while reducing regulatory T cells and myeloid‐derived suppressor cells. Immunosuppressive and glycolytic metabolites progressively declined, correlating with reduced tumor burden. Overall, DSTAP‐R848 enhances chemotherapy efficacy by reshaping immune and metabolic pathways and promoting durable anti‐tumor immunity in PC.

## Graphical abstract

Peritoneal carcinomatosis is a hard‐to‐treat spread of abdominal cancers that often returns after therapy. Nanoparticles were developed to retain an immune‐boosting drug in the abdomen. When combined with chemotherapy in mice, this approach eliminated tumors in many cases and prevented recurrence, helping the immune system mount a stronger, longer‐lasting anti‐cancer response.

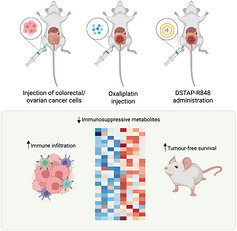

## 1 Introduction

Peritoneal carcinomatosis (PC) is an indication that arises when different gastrointestinal or gynecologic cancers metastasize to the peritoneal lining [1]. It is characterized as a disease with high recurrence rates of up to 76% within 5 years, despite aggressive treatments including cytoreductive surgery and hyperthermic intraperitoneal chemotherapy [2, 3]. Therefore, novel treatment methods are needed to address this disease.

While immunotherapy has emerged as a potential treatment method, the immunosuppressive tumor immune microenvironment (TIME) of PC severely hinders its efficacy [4]. Typically, the peritoneal environment consists of immune cells such as macrophages, dendritic cells (DCs), eosinophils, monocytes, B‐cells, innate lymphoid cells and others [5]. These immune cells work in tandem to maintain a healthy immune microenvironment in an individual. However, the presence of peritoneal tumors can polarize the immune cells in the environment to an immunosuppressive state, resulting in a poor prognosis [6]. This pro‐tumoral TIME consists of increased T regulatory cells (Tregs), M2 macrophages, and myeloid‐derived suppressor cells (MDSCs) [6, 7]. The presence of these immunosuppressive cells encourages immune evasion by the tumor and subsequently results in an increased resistance to the currently available immunotherapy, immune checkpoint inhibitors (ICIs) [8]. Therefore, a strategy to reverse this immunosuppression and repolarize the TIME to an immune‐active state, with increased tumor infiltration of DCs, CD8 + and CD4 + T cells, natural killer (NK) cells, and M1 macrophages, could eliminate the tumor cells and augment the efficacy of other treatments, including ICIs.

Immune modulators such as cytokines and imidazoquinolines have been used to modulate the TIME, with an example being Resiquimod (R848) [9]. By activating the toll‐like receptors (TLR) 7 and 8 and signaling through the myeloid‐differentiation factor 88 (MyD88) pathway, type I interferons (IFNs) are secreted resulting in increased DC activation, T cell priming and improved anti‐tumor response [10]. As overactivation of the immune system can result in cytokine storm, the development of a nanoparticle formulation to deliver R848 could localize inflammatory effects and produce a safer and more effective treatment.

In this study, cationic liposomes were developed using 1,2‐stearoyl‐3‐trimethylammonium‐propane chloride (DSTAP), cholesterol and 1,2‐Distearoyl‐sn‐glycero‐3‐phosphorylcholine (DSPC) to localize R848 to the peritoneal cavity. This treatment was combined with Oxaliplatin (Oxa), an immunogenic cell death (ICD) inducer, for treating two different models of peritoneal metastasis originating from colorectal cancer and ovarian cancer. Peritoneal fluid after different treatments was filtered and added to naïve splenocytes to demonstrate the polarization of immune cells upon exposure to different TIME. The temporal modulation of the TIME across different dosages was then assessed via flow cytometry of tumor infiltrating immune cells. Lastly, the peritoneal fluid of mice treated with Oxa only and Oxa+DSTAP‐R848 was metabolically assayed at different timepoints throughout treatment with Oxa only and Oxa+DSTAP‐R848, to observe the metabolic profiles and presence of immunosuppressive metabolites.

## 2 Materials and Methods

### 2.1 Chemicals and Reagents

DSTAP and DSPC were purchased from Avanti Polar Lipids (Alabaster, AL, USA). Acetic acid, cholesterol, dimethyl sulfoxide (DMSO), Dulbecco's modified Eagle Medium (DMEM), fetal bovine serum (FBS), penicillin and streptomycin and sodium acetate were purchased from Sigma‐Aldrich (St. Louis, MO, USA). Fluorescence‐activated cell‐sorting buffer, red blood cell lysis buffer, and antibodies were purchased from Thermo Fisher Scientific (Toronto, ON, Canada).

### 2.2 Liposome Preparation

50 mg/mL stocks of cholesterol and DSPC lipids were made in chloroform and a 50 mg/mL stock of DSTAP lipid was dissolved in chloroform/methanol (5:1, *v/v*). These were then mixed at a fixed molar ratio of 2:1:0.8, DSPC, cholesterol, and DSTAP. A thin film containing a total of ∼55 mg of lipid was then prepared using rotary evaporation. Afterward, this film was hydrated using 1 mL of 300 mm ammonium sulphate and then extruded at 65°C through a series of polycarbonate membranes starting from 400 to 200 nm and then finally to 100 nm using the Mini‐Extruder (Avanti, AL, USA). Monodispersed liposomes were then characterized by their size, polydispersity index (PDI) and zeta potential (ZP) using the Zetasizer Nano ZS (Malvern Instruments Ltd, Malvern, UK). Afterward, liposomes were dialyzed against 100 mm sodium acetate buffer (pH 5.5) for 24 h for drug loading.

### 2.3 Resiquimod Loading Into DSTAP‐Liposomes

Ultra‐high‐performance liquid chromatography (UPLC) was used to determine the total lipid content for drug loading. Resiquimod was solubilized in DMSO at 40 mg/mL and this mixture was then added to the liposomes at a drug to lipid ratio of 1:5 (w/w). This mixture was incubated for 60°C for 30 min and then cooled on ice for 5 min. To remove the unencapsulated Resiquimod, particles were dialyzed against 5% (w/v) glucose solution for 24 h. Total drug concentration was then determined by UPLC and particle characterization of DSTAP‐R848 was performed using the Zetasizer Nano ZS.

### 2.4 UPLC

To assess the lipid content or drug content of liposomes, these particles were dissolved in methanol (1:100 v/v) and mixed via pipetting.10 µL of the sample was injected into a Waters ACQUITY UPLC H‐Class system (Milford, MA, USA) and separated on a Waters Acquity BEH‐C18 column (particle size 1.7 µm, inner diameter 2.1 mm and length 50 mm) at a flow rate of 0.3 mL/min. The mobile phase of three different solvents at a varying gradient and are listed as follows: solvent A (0.1% trifluoroacetic acid (TFA) in water, v/v), solvent B (0.1% TFA in methanol, v/v) and solvent C (0.1% TFA in isopropanol, v/v). The gradient applied was 1.0 min: A/B/C (95/5/0), 2.0 min: A/B/C (5/95/0), 6.5 min: A/B/C (5/65/30), 7.1 min: A/B/C (95/5/0), 9.0 min: A/B/C (95/5/0). An evaporative light scattering detector (ELSD) was used to detect the sample and the data was analyzed using the Empower 3.0 software (Waters).

### 2.5 TEM Imaging

TEM images were captured with a Tecnai G20 at an accelerating voltage of 200 kV. The formulation was diluted to 0.4 mg/mL with distilled water and stained with 2% uranyl acetate. This was then deposited onto 400‐mesh Formvar‐coated carbon TEM grids. Particle images were then analyzed using ImageJ.

### 2.6 Stability Experiments

To determine stability of DSTAP‐R848 at body temperature, 100 µL of formulation was added to 2 mL 5% glucose. Particles were sized and characterized using the Zetasizer Nano ZS (Malvern Instruments Ltd, Malvern, UK) at 0, 1, 4, 6, and 24 h time points. To determine storage stability of DSTAP‐R848 at 4°C, particles were sized using the Zetasizer Nano ZS (Malvern Instruments Ltd, Malvern, UK) at 0, 7, 9, 12, and 14 days. Drug encapsulation was determined using UPLC using the methods described above. Prior to analysis, particles were taken out of storage and dialyzed against 5% glucose to remove any unencapsulated drug.

### 2.7 Ethical Statement

All animal experiments were performed in accordance with institutional guidelines of the University of British Columbia (UBC) under approved protocols #A22‐0141 and #A22‐0114.

### 2.8 Mice

For all studies using the CT26 models and ID8 models, female Balb/c mice (16–19 g, 5–8 weeks old, RRID: MGI:2161072) and female C57Bl/6 (16–19 g, 5–8 weeks old, RRID: IMSR_JAX:000664) respectively, were purchased from Charles River Laboratories (Wilmington, MA) and housed according to the institutional guidelines of UBC. Food and water were provided ad libitum.

### 2.9 Peritoneal Metastasis Model Development

For the peritoneal metastasis model developed using colorectal cancer cells, CT26 cells (RRID: CVCL_7254) were obtained from the American Type Cell Collection (Gaithersburg, ML, USA) and propagated in DMEM supplemented with 10% FBS and 1% penicillin/streptomycin. Mice were then inoculated with 2 × 10^5^ cells per mouse intraperitoneally (IP) to establish the model. For the model developed using ovarian cancer cells, ID8 cells (RRID: CVCL_IU14) were obtained from Dr. Julian Lum's lab from the University of Victoria, BC, Canada. These cells were also propagated in the same media. To establish the model, 5 × 10^6^ cells were injected peritoneally per mouse.

### 2.10 Cytokine Quantification

A single dose of DSTAP‐R848 at 4 mg/kg was given IP to mice with peritoneal metastasis of colorectal cancer 5 days after inoculation and 10 days after inoculation to mice with peritoneal metastasis of ovarian cancer. 1 h after injection, peritoneal fluid was taken out from the cavity and IFN‐α was measured using the eBioscience ELISA kits (Thermo Fisher, Toronto, ON, Canada) following the manufacturer's instructions.

### 2.11 In Vitro ATP assay

To quantify extracellular ATP release following drug treatment, CT26 or ID8 cell lines were seeded at a density of 1 × 10^4^ cells per well in 96‐well plates using their respective complete culture media, as described above. Cells were subsequently treated with either PBS or free Oxaliplatin at indicated concentration in the range of 2 to 200 µm.

Following adding treatment to the wells, the RealTime‐Glo Extracellular ATP Assay reagent (Promega, USA) was added to each well according to the manufacturer's instructions. Plates were incubated at 37°C, and luminescence was continuously monitored from 10 to 24 h after addition of the ATP Assay reagent. The signal was monitored using a CLARIOstar microplate reader (BMG LabTech, Germany). Luminescence measurements were recorded at 30‐min intervals.

### 2.12 In Vitro CRT Quantification Assay

Cells were seeded in 12‐well plates at a density of 2 × 10^5^ cells per well and incubated for 24 h at 37°C under 5% CO_2_ to allow for cell attachment. Cells were then treated with PBS, or oxaliplatin at the concentrations indicated (2 to 200 µm) and incubated overnight for 16 h. Following treatment, the medium was removed, and cells were washed once with pre‐warmed complete culture medium before being further incubated for an additional 8 h.

Cells were subsequently detached using TrypLE Express Enzyme (Gibco, USA), collected, and stained with Zombie Aqua Fixable Viability Dye (BioLegend, USA; 1:1000 dilution). After washing with cold FACS buffer, cells were stained with Alexa Fluor 647–conjugated anti‐calreticulin antibody (AB_196159, Abcam, UK; 1:200 dilution) or the corresponding Alexa Fluor 647–conjugated rabbit IgG isotype control (AB_199093, Abcam, UK, 1:200 dilution). Flow cytometric analysis was performed using a BD Symphony A1 Analyzer (BD, USA).

### 2.13 High‐Mobility Group Box 1 (HMGB‐1) Enzyme‐Linked Immunoassay (ELISA)

Cells were seeded at a density of 1 × 10^5^ cells per well in 24‐well plates and incubated for 24 h at 37°C with 5% CO_2_ to allow for cell attachment. The following day, cells were treated with varying concentrations of the test compounds (PBS, Oxa 2, 20, and 200 µm) prepared in complete culture media. After 24 h, cell culture supernatants were collected, and 10 µL of each sample was added to a commercial HMGB1 ELISA plate (IBL International, ST51011). Standards and blank controls were included on each plate and prepared according to the manufacturer's protocol. Plates were incubated at 37°C for 2 h, washed, and then incubated with the provided enzyme conjugate for an additional 2 h at room temperature. Colour development was initiated by adding substrate solution and allowing the reaction to proceed for 30 min before adding stop solution. Absorbance was measured at 450 nm using a CLARIOstar microplate reader (BMG LabTech, Germany).

### 2.14 Efficacy Study

For the colorectal cancer model, animals were randomized and mice (*n* = 10 per group) were injected with 6 mg/kg Oxaliplatin IP 4 days post inoculation. On Days 5, 7, 9, 11, 13, and 15, mice were given 2 mg/kg of DSTAP‐R848 IP or saline only. Survival was then monitored for 160 days. If mice reached a clinical health score of 4 or higher, they would be humanely euthanized. Mice that survived >160 days would be rechallenged using CT26 and 4T1 cells subcutaneously and monitored for tumor development. For the ovarian cancer model, animals were randomized and mice (*n* = 10 per group) were injected with 6 mg/kg Oxaliplatin IP 10 days post inoculation. On Days 11, 13, 15, 17, 19, and 21, mice were given 2 mg/kg DSTAP‐R848 IP. Survival was monitored for 160 days, and humane euthanasia was done for mice presenting with a clinical health score of 4 or higher. Surviving mice would be rechallenged using ID8 and B16F10 cells subcutaneously and monitored for tumor development. When anti‐PD‐1 was included in the treatment scheme, an IP injection of 200 µg was given concurrently with DSTAP‐R848 or saline and the same treatment scheme was used.

### 2.15 Tumor Immune Microenvironment Characterization

Mice (*n* = 9‐10 per group for Oxa+DSTAP‐R848 treated and *n* = 3‐6 for Oxa only control treated) were inoculated with 2 × 10^5^ CT26 cells IP. On day 7, a single dose of 6 mg/kg Oxaliplatin was given IP and then on days 8, 10, 12, 14, 16, and 18, DSTAP‐R848 or saline was given IP at 2 mg/kg. On days 10, 12, and 18, mice were taken down and their tumors excised. For flow cytometry analysis, tumor tissue was digested using 1X collagenase/hyaluronidase (StemCell Therapeutics, Vancouver, BC, Canada) in RPMI for 30 min at 37°C. Tumors were then mashed through a 0.45 µm cell strainer to isolate single cells for flow cytometry. The following antibodies were used for analysis: CD45‐Alexa Fluor 700 (AB_2734986), CD206‐eFluor 450 (AB_2762721), F4/80‐APC (AB_2784648), CD11c‐Alexa Fluor 488 (AB_10373244), Gr‐1‐PE (AB_2651871), CD11b‐eFluor 450 (AB_1582237), CD3‐APC‐eFluor 780 (AB_1272181), CD4‐PE (AB_465510), CD25‐Alexa Fluor 488 (AB_763472), FoxP3‐eFluor660 (AB_11218868), NK1.1‐APC (AB_313397), CD8‐APC (AB_469335), and IFN‐γ‐Alexa Fluor 488 (AB_469932). Antibodies were either obtained from ThermoFisher Scientific (Toronto, ON, Canada) or Biolegend (San Diego, CA). For intracellular proteins, the Foxp3/Transcription Factor Staining Buffer Set (Cat. No. 00‐5523‐00) was used from ThermoFisher Scientific (Toronto, ON, Canada). The specific gating strategy is available in Figure S8.

### 2.16 Splenocyte Polarization Characterization

Peritoneal fluid was collected from mice with CT26 peritoneal metastasis treated with Oxa only or Oxa+DSTAP‐R848 for a total of six doses (*n* = 3 per group). This fluid was flash frozen in liquid nitrogen and stored at −80°C until use. To perform the assay, spleens were taken from naïve mice (*n* = 4) and broken down into single cells by mashing them through a 0.45 µm cell strainer. Splenocytes were then pooled and plated at 2 × 10^5^ cells per well in RPMI media with 10% FBS and 1% penicillin/streptomycin and 100X diluted peritoneal fluid from each treatment (*n* = 3 per group) in a 96‐well TC treated flat‐bottom plate (Cat. #701001, Luna Nanotech, Toronto, Ontario). Splenocytes were incubated for 24 h at 37°C with 5% CO_2._ Cells were then collected for flow cytometry analysis using the panel and gating strategies described above.

### 2.17 Metabolomics

Mice (*n* = 3 per group) were inoculated with 2 × 10^5^ CT26 cells IP. On day 7, a single dose of 6 mg/kg Oxaliplatin was given IP and then on days 8, 10, 12, 14, 16, and 18, DSTAP‐R848 or saline was given IP at 2 mg/kg. On days 10, 12, and 18, groups of mice (*n* = 3 per group) were euthanized and their peritoneal fluid or ascites collected for metabolomic analysis using mass spectrometry. Samples were flash frozen in liquid nitrogen and stored at −80°C until analysis. Data was analyzed using MetaboAnalyst 6.0 [11].

### 2.18 Assay of Amino Acids and Amines for Metabolomics

Assay of amino acids and amines used a chemical derivatization – ultra high‐performance liquid chromatography – multiple‐reaction monitoring mass spectrometry (UPLC‐MRM/MS) method that we described previously [12], with necessary adaptations and modifications.

A cocktail of 50 isotope‐labelled amino acids and amines was prepared in 50% aqueous acetonitrile and used as an internal standard (IS) solution. A standard‐substance solution containing all the measured amino acids, amines and metabolic intermediates of amino acid anabolism and degradation was prepared in water, at a concentration of 100 µm for each compound. This solution was serially diluted with the same solvent to produce ten calibration solutions.

20 µL of each ascites sample, with and without 50‐fold dilution, and 20 µL of each calibration solution, were mixed in turn with 20 µL of the IS solution, 120 µL of 10‐mm dansyl chloride solution and 30 µL of a borate buffer at pH 9. The mixtures were incubated at 50°C for 30 min and then cooled down on ice for 5 min, followed by centrifugation at 21 000 g and 5°C for 10 min. 6 µL aliquots of the clear solutions were injected to run UPLC‐MRM/MS on an Agilent 1290 UPLC system coupled to an Agilent 6495C triple‐quadrupole (QQQ) mass spectrometer equipped with an electrospray ion source and operated with positive‐ion detection. A C18 column (2.1^*^150 mm, 1.8 µm) was used for chromatographic separation with a mobile phase composed of 0.1% formic acid in water (A) and 0.1% formic acid in acetonitrile – isopropanol (1:1, v/v) (B) as binary solvents for gradient elution under optimized operation conditions.

### 2.19 Assay of Phosphate‐Containing Metabolites for Metabolomics

A standard solution containing standard substances of all the phosphate‐containing metabolites was prepared in 80% acetonitrile at 50 µm for each compound. This solution was diluted step by step in the same solvent to generate nine calibration solutions. Next, 20 µL of each ascites sample was mixed with 80 µL of acetonitrile. The mixtures were vortexed for 1 min, sonicated for 1 min and then centrifuged at 21 000 g and 5°C for 10 min. 20 µL of the clear supernatant from each sample or 20 µL of each calibration solution was mixed with 80 µL of an IS solution containing isotope‐labelled AMP, ATP, GMP, GTP, fructose‐6‐phosphate, NAD and nicotinic acid. 10 µL aliquots of the resultant solutions were injected into a C18 column (2.1^*^100 mm, 1.8 µm) to run UPLC‐MRM/MS on a Waters Acquity UPLC system coupled to a Sciex QTRAP 6500 Plus mass spectrometer equipped with an electrospray ion source and operated with negative‐ion detection. A mobile phase composed of a tributylamine buffer solution (A) and acetonitrile (B) was used for binary‐solvent gradient elution under optimized operation conditions.

### 2.20 Assay of Fatty Acids and Low‐Molecular‐Weight Sugars for Metabolomics

Assay of short‐chain to long‐chain fatty acids and low‐molecular weight sugars was conducted using a chemical derivatization – UPLC‐MRM/MS method, which was modified and adapted from the procedures that we described previously [12, 13, 14]. An IS solution containing 9 isotope‐labelled carboxylic acids [15] and ^13^C_6_‐glucose was prepared in methanol. A stock solution of all the measured fatty acids and sugars was prepared and serially diluted in the IS solution. Next, 20 µL of each ascites sample or each calibration solution was mixed with 20 µL of the IS solution, 40 µL of 200‐mm 3‐nitrophenylhydrazine solution and 40 µL of 150‐mm EDC‐6% pyridine solution. The mixtures were incubated at 40°C for 60 min. After cool‐down and centrifugation at 21 000 g and 5°C for 10 min, 5 µL aliquots were injected into a C18 column (2.1^*^50 mm, 1.8 µm) to run UPLC‐MRM/MS on an Agilent 1290 UPLC system coupled to an Agilent 6495B triple‐quadrupole (QQQ) mass spectrometer equipped with an electrospray ion source and operated with negative‐ion detection. For chromatographic separation, a mobile phase composed of 0.01% formic acid in water (A) and 0.01% formic acid in acetonitrile – isopropanol (1:1, v/v) (B) was used as binary solvents for gradient elution under optimized operation conditions.

### 2.21 Assay of Other Metabolites for Metabolomics

A standard solution that contained standard substances of the other metabolites was prepared in 70% acetonitrile. This solution was serially diluted in the same solvent to generate nine calibration solutions. An IS containing 20 isotope‐labelled nucleosides, carnitines and thiol metabolites was prepared in 20‐mm N‐ethylmaleimide aqueous solution. For sample preparation, 30 µL of each ascites sample was mixed with 70 µL of acetonitrile. The mixture was vortexed and placed on ice for 30 min, followed by centrifugation at 21 000 g and 5°C for 10 min. 20 µL of the clear supernatant from each sample or 20 µL of each calibration solution was mixed with 80 µL of the IS solution. 10 µL aliquots of the resultant solutions were injected to run UPLC‐MRM/MS on an Agilent 1290 UPLC system coupled to an Agilent 6495C QQQ mass spectrometer equipped with an electrospray ion source and operated with positive‐ion detection. A C18 column (2.1^*^100 mm, 1.8 µm) was used for chromatographic separation and a mobile phase composed of an HFBA solution and (A) and acetonitrile (B) was used for binary‐solvent gradient elution under optimized operation conditions for both chromatographic separation and MRM/MS detection.

In the above assays, the LC‐MRM/MS data acquired on the Agilent UHPLC‐QQQ systems were recorded and processed using the Agilent MassHunter 10.0 software suite. The LC‐MRM/MS data acquired on the Waters Acquity UPLC – Sciex QTRAP 6500 Plus system were recorded and processed using the Sciex OS 2.0 software suite. Linearly regressed calibration curves of individual metabolites were constructed with the data acquired from the calibration solutions in each of the assays. Concentrations of the metabolites detected in the samples were calculated by interpolating the calibration curves with the analyte‐to‐IS peak area ratios of individual metabolites, which were measured from the sample solutions.

### 2.22 Metabolomics Data Analysis

For the metabolomics data, one factor statistical analysis was performed using MetaboAnalyst 6.0. Data was normalized by sum, log (base 10) transformed, and pareto‐scaled prior to analysis. Principle component analysis (PCA), heat map, variable importance in projection (VIP) scores and volcano plots were all determined using the MetaboAnalyst 6.0 webtool with three biological replicates sampled per metabolite for significance testing. Heatmap dendrograms were constructed using Ward's method to cluster Euclidean distances among samples. Columns were grouped by treatment and timepoint for visualization.

### 2.23 Statistical Analysis

All data are expressed as mean ± SEM. Statistical analysis was conducted with the two‐tailed unpaired *t*‐test for two‐group comparison or one‐way ANOVA followed by the Tukey's multiple comparison test for three or more groups, or a log‐rank (Mantel‐Cox) test for survival data, using GraphPad Prism 9 (San Diego, CA). A difference with *p* < 0·05 was considered to be statistically significant.

## 3 Results

### 3.1 DSTAP‐R848 Retained in the Peritoneal Cavity over 24 h and Extended Survival in Mouse Models of Peritoneal Metastasis When Combined With Oxaliplatin

**FIGURE 1:**
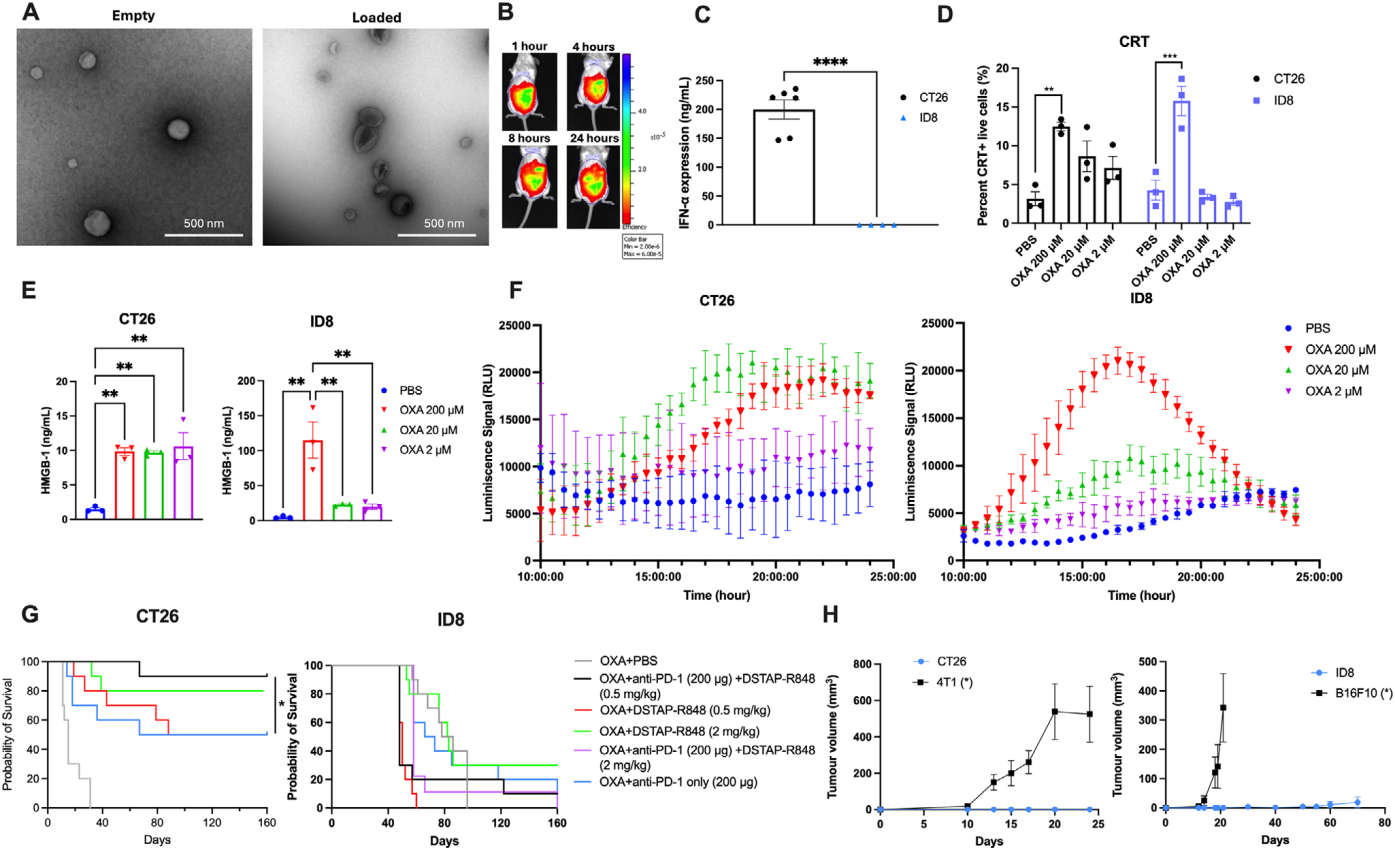
Characterization of DSTAP‐R848 and efficacy in two PC models. (A) Empty vs. loaded DSTAP liposomes visualized under TEM at 30 000x magnification. (B) IVIS imaging of mice after intraperitoneal injection with DiR‐labeled DSTAP liposomes at 0·3 µg DiR/mouse. (C) IFN‐α levels in the peritoneal fluid of mice with PC of CT26 or ID8, 1 h post injection of DSTAP‐R848. Data are presented as mean ± standard error mean (SEM). Statistics were performed using an unpaired *t*‐test (^****^, *p* < 0·0001). (D) Quantification of CRT+ live cells in the CT26 and ID8 cell line after exposure to Oxa at 2, 20, and 200 µm. Data are presented as mean ± standard error mean (SEM). Statistics were performed using one‐way ANOVA with Tukey's post‐hoc test (^***^, *p* < 0·001, ^**^, *p* < 0·01). (E) Quantification of HMGB‐1 in CT26 and ID8 cells after exposure to Oxa at 2, 20, and 200 µm. Data are presented as mean ± standard deviation (SD). Statistics were performed using one‐way ANOVA with Tukey's post‐hoc test (^**^, *p* < 0·01). (F) ATP release over time in CT26 and ID8 cell lines after exposure to Oxa at 2, 20, and 200 µm. (G) Survival of mice with PC of CT26 or ID8 across different treatment groups throughout 160 days. Median survival CT26: Oxa+anti‐PD‐1 only, 113.5 days, Oxa+DSTAP‐R848 (0.5 mg/kg), 124 days, and Oxa+PBS, 15 days. Median survival ID8: Oxa+PBS, 82 days. Oxa+anti‐PD‐1+DSTAP‐R848 (0.5 mg/kg), 48 days. Oxa+DSTAP‐R848 (0.5 mg/kg), 50 days. Oxa+DSTAP‐R848 (2 mg/kg), 82.5 days. Oxa+anti‐PD‐1+DSTAP‐R848 (2 mg/kg), 58 days. Oxa+anti‐PD‐1 only, 66 days. Statistics were performed using the log‐rank (Mantel‐Cox) test. (^*^, *p* < 0.05) (H) Rechallenge graphs and tumor sizes of original and new cancer cell lines in mice cured of PC of CT26 or ID8. Statistics were performed using an unpaired *t*‐test (*, *p* < 0.05).

Cationic liposomes were developed using a commercially available monovalent cationic lipid DSTAP, DSPC and cholesterol. These nanoparticles were then actively loaded with R848 using the SALT method [16], followed by removal of unencapsulated drug. Through dynamic light scattering (DLS), DSTAP‐R848 was found to be 159·03 ± 1·03 nm in size with a zeta potential of 39·51 ± 5·61 mV. The encapsulation efficiency was 65·61 ± 5·43%. With the positive charge and small size, its characteristics favor uptake by cells with low toxicity, as previously demonstrated [17]. The incorporation of the DSTAP lipid also showed improved peritoneal retention compared to neutral and negatively charged liposomes [17]. The morphology of empty DSTAP liposomes and R848‐loaded DSTAP liposomes was visualized through transmission electron microscopy (Figure 1A). An electron‐dense core was revealed in DSTAP‐R848, indicating the formation of R848 crystals within the inner aqueous phase (Figure S1). Additionally, DSTAP‐R848 was found to remain below 190 nm in size with a PDI below 0.200 at 37°C over 24 h, allowing for uptake. This formulation was also found to be stable in both size and drug encapsulation in storage at 4°C for at least 2 weeks Figure S2). When incorporated with 1,1'‐Dioctadecyl‐3,3,3',3'‐Tetramethylindotricarbocyanine iodide (DiR) in the liposomal bilayer, injected peritoneally and assessed over a 24‐h period, DSTAP liposomes were found to be retained in the abdominal cavity with minimal systemic exposure (Figure 1B). When a single dose of DSTAP‐R848 at 4 mg/kg was intraperitoneally injected into mice bearing peritoneal metastases of ID8 ovarian cancer or CT26 colorectal cancer cells, high levels of IFN‐α were measured in the peritoneal fluid of the CT26 model but not in the ID8 model an hour after administration (Figure 1C). This suggested that the ID8 model could potentially have a colder TIME compared to the CT26 model.

Next, we assessed whether Oxa could induce ICD of ID8 and CT26 cells. Cells were treated with Oxa at 2, 20, and 200 µm, and ICD markers including calreticulin (CRT), HMGB‐1 and ATP were quantified. As shown in Figure 1D, CRT levels detected at the cell surface were significantly higher after treatment with Oxa at 200 µm in both cell lines, but not at lower concentrations. For HMGB‐1, all doses of Oxa induced significantly higher levels of the protein in the supernatant of the CT26 culture while only the highest dose was effective in the ID8 culture Figure 1E). ATP kinetics were measured via luminescence (Figure 1F), and in both cell lines, Oxa at 20 and 200 µm significantly increased the secreted ATP production, while the effect of Oxa at 20 µm in ID8 cells was only moderate. The ICD assays suggested that Oxa induced ICD in both cell lines with a stronger effect in CT26 compared to ID8.

We hypothesized that when DSTAP‐R848 was administered in a treatment scheme with Oxa, the induced ICD would release tumor‐associated antigens to train the activated immune cells to act against the tumor. We then tested this hypothesis by monitoring survival of animals bearing peritoneal metastases of CT26 or ID8 cells after different treatments. Treatment was initiated at its respective timepoints for each model to mimic the clinical conditions after debulking surgery where minimal residual tumor burden is observed. In the CT26 model, mice treated with Oxa+PBS reached experimental endpoints before 30 days‐post tumour implantation. However, mice treated with Oxa+DSTAP‐R848 at 0·5 and 2 mg/kg displayed a 50% and 80% response rate, respectively (Figure 1G). We further hypothesized that Oxa+DSTAP‐R848 treatment would result in additive effects with the immune checkpoint inhibitor anti‐PD‐1. When given separately, Oxa+DSTAP‐R848 (0·5 mg/kg) or Oxa+anti‐PD‐1 yielded a response rate of 50%, while the combination cured 90% of mice in the CT26 model (Figure 1G). In the ID8 model, the OXA+PBS control all eventually succumbed to disease by Day 90 whereas the Oxa+DSTAP‐R848 (2 mg/kg) group retained a cure rate of 30%. Combining Oxa with a lower dose of DSTAP‐R848 (0·5 mg/kg) was ineffective in the ID8 model without any long‐term survivors. Anti‐PD‐1 did not appear to have any synergy with DSTAP‐R848, and in fact the combination resulted in a potentially antagonizing effect with the cure rate dropping to only 10% (Figure 1G).

In both groups, surviving mice were rechallenged with the original cell lines for 60–65 days with no recurrence in the CT26 model and one out of three mice developing a small, slow growing tumor in the ID8 model (Figure 1H). The inferior efficacy of Oxa‐DSTAP‐R848 in ID8 compared to CT26 could be attributed to its decreased response to Oxa and DSTAP‐R848 (ICD and IFN‐α, respectively). As the success of the treatment depended on the presence of ICD and the induction of IFNs and cytokines, we were interested in understanding how the TIME was affected. To understand its mechanism, the CT26 model was chosen due to its higher treatment response rate.

### 3.2 Naïve Splenocytes Were Polarized to Immunosuppressive and Activated Phenotypes When Exposed to Peritoneal Fluid With Oxa Only vs. Oxa+DSTAP‐R848 Treatment, Respectively

**FIGURE 2:**
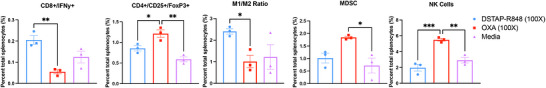
Changes in splenocyte composition after 24‐h exposure to filtered peritoneal fluid from Oxa+DSTAP‐R848 treated mice, Oxa only treated mice or culture media (*n* = 3 per group). Data are presented as mean ± standard error mean (SEM). Statistics were performed using a one‐way ANOVA with Tukey's post‐hoc test (^***^, *p* < 0·001, ^**^, *p* < 0·01, ^*^, *p* < 0·05).

To determine the impact of the TIME on the polarization of naïve immune cells, peritoneal fluid and ascites was taken from Oxa only and Oxa+DSTAP‐R848 mice after the sixth dose of DSTAP‐R848 or saline treatment. All samples were filtered to remove residual cells and incubated with naïve splenocytes for 24 h. In Figure 2, splenocytes incubated with fluid from OXA+DSTAP‐R848‐treated mice had 3·73‐fold higher percentages of CD8+/IFN‐γ+ cells compared to the Oxa group. This hinted that an immune‐active microenvironment was generated by OXA+DSTAP‐R848. The splenocytes exposed to the peritoneal fluid of Oxa‐treated mice had significantly higher percentages of Treg cells (CD4 +/CD25 +/FoxP3 +), suggesting Oxa only treatment skewed the TIME toward immunosuppression. Splenocytes conditioned with the fluid from the Oxa+DSTAP‐R848 group also displayed a higher M1/M2 ratio compared to the Oxa only control, supporting the idea that the peritoneal environments of DSTAP‐R848 treated mice may have enhanced the proliferation or polarization of anti‐cancer immune cells. MDSC percentage in the Oxa sample increased, while that in the Oxa+DSTAP‐R848 group showed no difference compared to the media control, indicating an immunosuppressive environment after Oxa only therapy. The increase in NK cells in the Oxa group was likely due to the higher presence of tumor‐associated factors in the Oxa only fluid. Overall, our data indicate that when naïve splenocytes were exposed to the Oxa+DSTAP‐R848‐treated environment, higher percentages of immune cells relating to anti‐cancer activity were detected. As factors in the TIME were found to polarize immune cells into different phenotypes, we sought to further investigate how Oxa+DSTAP‐R848 modulates this process at different time points throughout treatment.

### 3.3 Oxa‐DSTAP‐R848 Treated CT26 Tumors Showed Decreased Treg and Increased IFN‐𝛄+ CD8+ T Cell Infiltration

**FIGURE 3:**
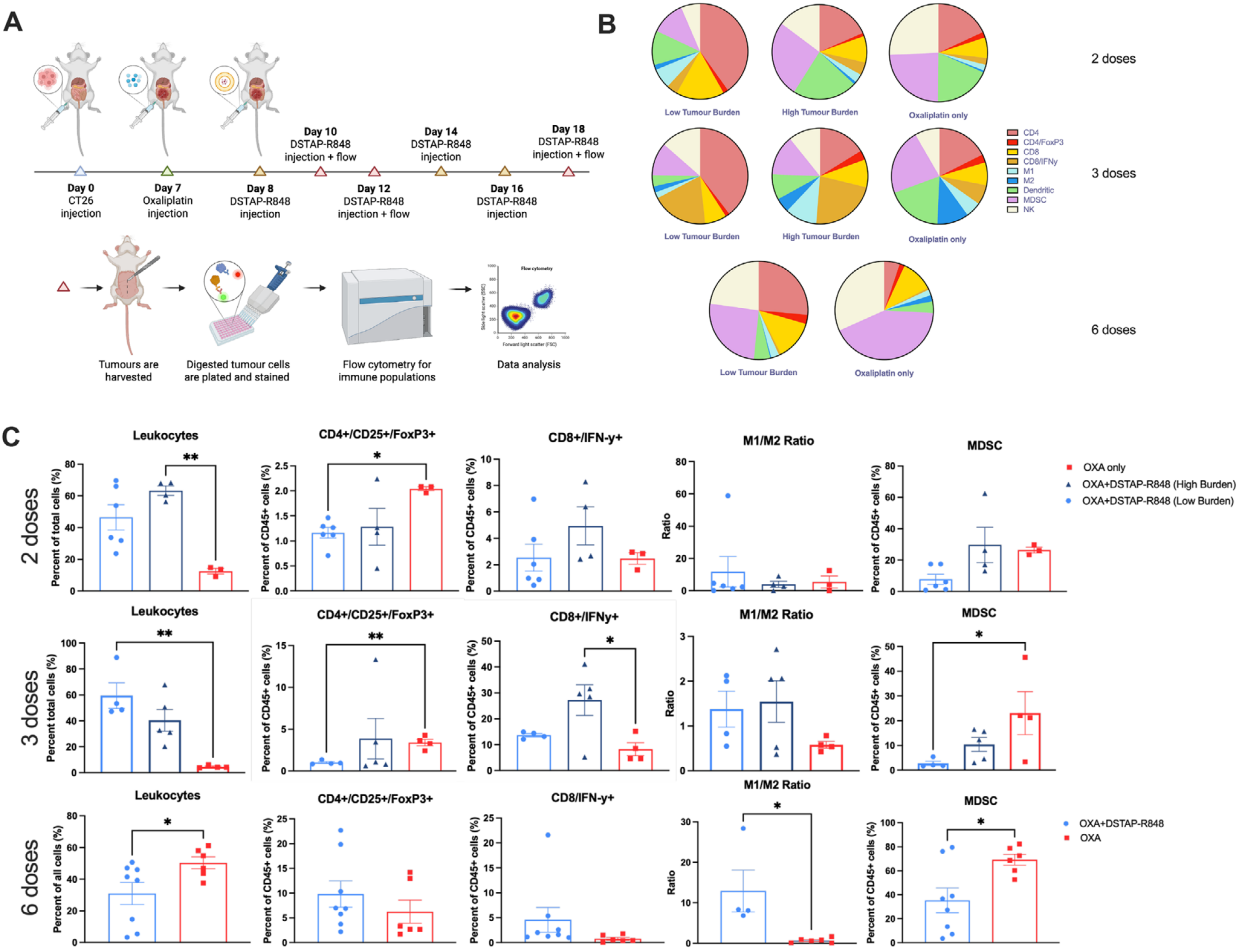
Temporal assessment of the TIME throughout treatments with DSTAP‐R848. (A) Treatment and sample harvest regimen. (B) Pie charts of different immune populations in each treatment group. (C) Percentage of leukocytes and various immune populations within the harvested tumors across time points. Data are presented as mean ± standard error mean (SEM). Statistics were performed using one‐way ANOVA and Tukey's post‐hoc test (^**^, *p* < 0·01, ^*^, *p* < 0·05).

To visualize the changes in immune composition across the doses, tumors from cohorts of treated and control mice were collected after the second (day ten), third (day twelve), and sixth doses (day eighteen) of DSTAP‐R848 (2 mg/kg), and processed for flow cytometry (Figure 3A). Tested populations included CD4+ T cells, T regulatory cells (CD4+/CD25+/FoxP3+), CD8+ T cells, activated cytotoxic CD8+ T cells (CD8+/IFN‐γ+), M1/M2 macrophages, DCs, MDSCs and NK cells. Mice were found to have either high or low tumor burdens upon harvest, with low burden (LB) mice being defined as having <5 tumor nodules, and high burden (HB) mice having >20 tumor nodules, no mice presented with nodule numbers between the two (Figure S3). At two doses, four out of ten mice were found to have HB and six had were classified as LB. At three doses, five out of nine mice were HB and the other four were LB. No HB mice were found after six doses due to tumor shrinkage. To visualize the tumor immune composition changes amongst groups, percentages of each immune population are represented in pie charts in Figure 3B. After two doses, HB mice had a 5·05‐fold increase in percentage of infiltrating leukocytes in the tumor compared to the control that had only received Oxa only (Figure 3C). At this time point, groups with a lower tumor burden had a 1·69‐fold reduction of percentage of infiltrating Treg cells whereas the HB mice had more variability. After the third dose, LB mice had a higher percentage of infiltrating leukocytes with a 13·05‐fold increase compared to the Oxa only control. The HB tumors also had a 9·98‐fold increase compared to the control, but this was not significant. This time point had the most significant difference in both Treg cells and activated IFN‐γ secreting cytotoxic CD8+ T cells, with a 3·72‐fold decrease in Treg in the LB mice compared to the Oxa control, and a 2·48‐fold increase in HB mice, respectively. LB mice had a 1·44‐fold increase in infiltration of IFN‐γ+/CD8+ T cells compared to the Oxa control, but this was not statistically significant. At this time point, MDSCs within the tumor were also significantly different between the LB group and Oxa control, with a 5·65‐fold decrease in the LB group compared to the control. At the six‐dose time point, groups were stratified into Oxa only and Oxa+DSTAP‐R848 groups as eight out of eight Oxa+DSTAP‐R848 treated mice displayed LB. Surprisingly, leukocytes were 1·63‐fold lower in the Oxa+DSTAP‐R848 group compared to the Oxa control, however no difference was found in the percentages of CD4+ T cells, Treg, CD8+ T cells, DCs, and NK cells between the two groups (Figure S4). However, the M1/M2 ratio was 18·8‐fold higher in the Oxa+DSTAP‐R848 group compared to the Oxa control and the MDSCs were 1·96‐fold lower comparatively, suggesting that the Oxa control tumors were still in an immunosuppressed state (Figure 3C).

Overall, response was not homogenous within the Oxa+DSTAP‐R848 treated groups at early stages of treatment. Mice were stratified into HB and LB groups at early doses, vs. the Oxa only control. Generally, both day ten and day twelve timepoints showed a decrease in immunosuppressive cells (Tregs and MDSCs), while three doses at day twelve were necessary for the induction of a significant infiltration of cytotoxic T cells. At the six dose timepoint at day eighteen, very few tumors were observed in the Oxa+DSTAP‐R848 treated group, and leukocyte infiltration was generally lower. However, the decreased percentages of MDSCs persisted compared to the Oxa only group and the M1/M2 macrophage ratio was much higher when treated with DSTAP‐R848, suggesting macrophage polarization. Oxa+DSTAP‐R848 treated lesions were likely shrinking compared to Oxa only treated ones, where tumor burden was continuing to increase.

### 3.4 Glycolysis and Tryptophan Metabolites Drove Differences Between Profiles of Oxa Only vs. Oxa+DSTAP‐R848 Treated Groups

**FIGURE 4:**
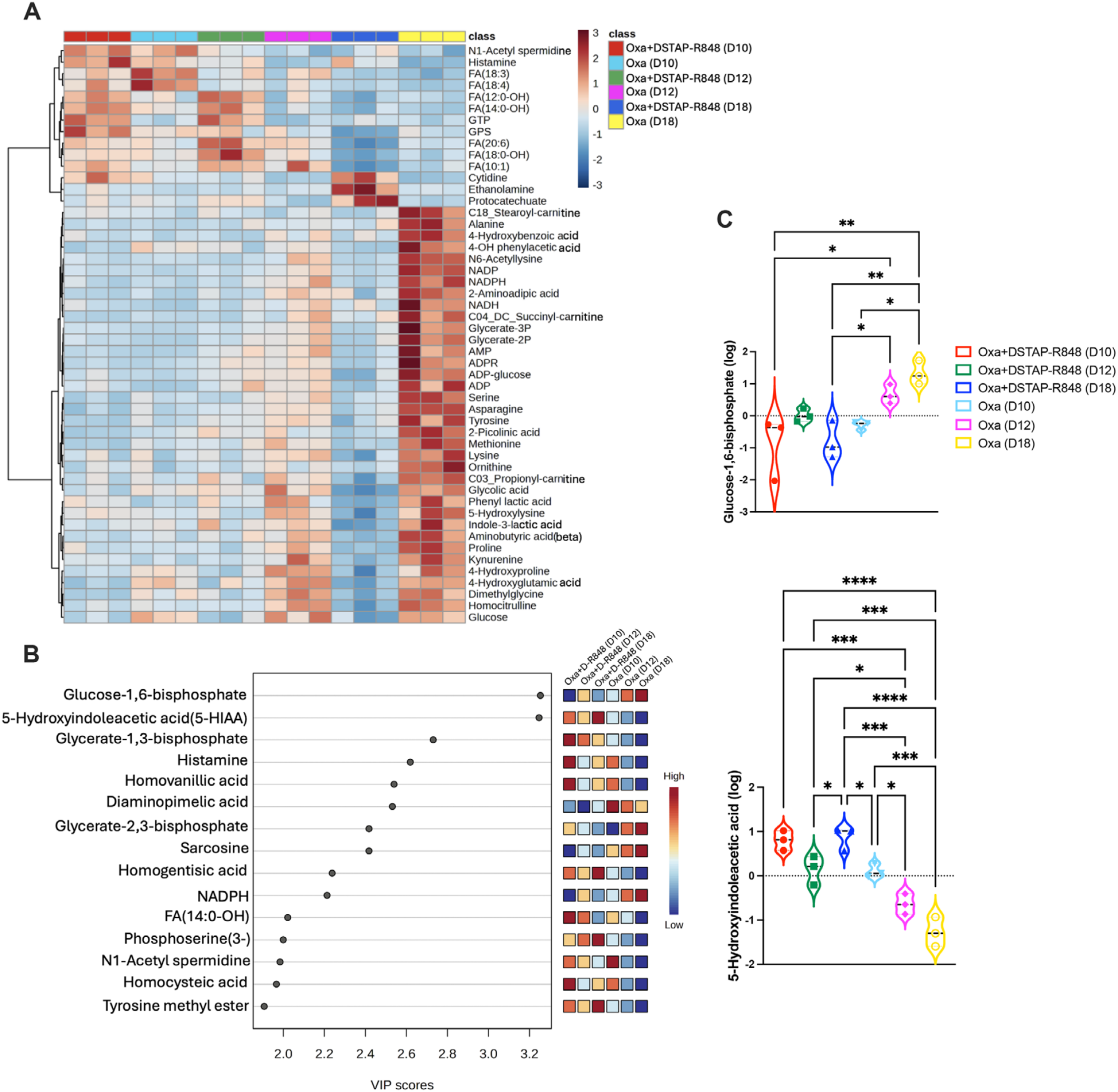
Metabolite heatmaps and Variable Importance in Projection (VIP) scores. (A) Heatmap of the normalized metabolite abundance. (B) VIP scores showing important metabolites driving the differences between groups. (C) Normalized log10 values of glucose‐1,6‐bisphosphate and 5‐hydroxyindoleacetic acid across different groups. Statistics were performed using a one‐way ANOVA with Tukey's post‐hoc test (^****^, *p* < 0·0001, ^***^, *p* < 0·001, ^**^, *p* < 0·01, ^*^, *p* < 0·05).

Temporal changes in tumor burden and immune cell subtypes after administration of DSTAP‐R848 suggested that there could also be metabolic changes that affected the immunosuppressive microenvironment. By assaying the metabolites, we can validate the levels of immunosuppression present in the TIME across different timepoints, as well as identify potential biomarkers that could inform on prognosis during treatment with immunotherapies. To profile these fluctuations, mice were intraperitoneally inoculated with CT26 cells and treated with the same regimen given to the groups in the flow cytometry cohort. Two hours after each dose, fluid was lavaged from the peritoneal cavity and metabolically profiled. Unsupervised hierarchical clustering of metabolomic profiles from the TIME revealed distinct, treatment and time‐dependent metabolic signatures (Figure 4A). Metabolite abundances were log‐transformed and normalized across samples prior to analysis. The heat map includes metabolites that were significantly altered across treatment groups and time points (ANOVA, *p* < 0·05). Samples organized by timepoints revealed the progressive divergence of metabolites with and without treatment. Earlier time points at day ten showed relatively conserved metabolic profiles and began diverging between Oxa only and Oxa+DSTAP‐R848 treatments on day twelve. Generally, immunosuppressive pathways were enriched in the Oxa only group with higher concentrations of kynurenine (Kyn) and ornithine. Additionally, high relative levels of NADPH were observed suggesting increased energy levels to sustain rapid growth. This was also found to be relevant to increased tryptophan metabolism (high Kyn, and alanine) and tyrosine biosynthesis (high hydroxyphenyl acetic acid), as well as the Warburg effect (high ADP and 3‐phosphoglyceric acid) in the Oxa only group (Figure 4A). The principal component analysis (PCA) plot showed clustering of treatment groups and timepoints based on metabolic profiles, revealing that metabolic profiles of Oxa only and Oxa+DSTAP‐R848 at day eighteen were most varied from one another (Figure S5). The PCA also grouped metabolic profiles of the Oxa only and Oxa+DSTAP‐R848 day ten or dose two and day twelve or dose three of DSTAP‐R848 time points together likely suggesting low levels of tumor‐associated metabolites at these time points. The Oxa day twelve group was grouped as a separate cluster and was seen to lean toward the Oxa day eighteen group, suggesting the accumulation of tumor‐associated metabolites as time progressed (Figure S5). The metabolic heatmap also reflected this difference with Oxa only groups increasing in tumor‐associated metabolite abundance. Within the Oxa+DSTAP‐R848 group, we observed higher levels of immunosuppressive metabolites like N1‐acetylspermidine near the beginning of treatment, which decrease by day eighteen. By mapping out the metabolites throughout treatment between the two groups, we can visualize the fluctuations as the cancer burden either increases or decreases with or without DSTAP‐R848 treatment.

Variable importance in projection (VIP) scores showed that the biggest metabolic drivers of the observed differences among these 6 groups were glucose‐1,6‐bisphosphate (Glc‐1,6‐BP) and 5‐hydroxyindoleacetic acid (5‐HIAA) (Figure 4B). Glc‐1,6‐BP is a byproduct of glycolysis and was increasing in concentration over time in the Oxa only control whereas its levels fluctuated in the DSTAP‐R848 treated group (Figure 4C). Both Oxa and Oxa+DSTAP‐R848 treated groups started with relatively similar levels of Glc‐1,6‐BP. After the third dose at day twelve, Oxa+DSTAP‐R848 groups had a 3·18‐fold increase in levels of the metabolite compared to the second dose at day ten, potentially due to the increase in tumor burden as well as immune activity. Figure 3C showed an increase in cytotoxic CD8+ T cell infiltration in HB mice which could also contribute to the levels of Glc‐1,6‐BP (Figure 4B). For the Oxa only group, a 10·98‐fold increase in Glc‐1,6‐BP was observed but this increase is more likely to be attributed to the increase in tumor burden as well as consequences of the Warburg effect from the tumors. After the sixth dose at day eighteen, the treated group saw a 4·16‐fold decrease of the metabolite from day twelve, which matched the decline in immune activity and tumor burden observed (Figures 3 and 4C). The trend of the Oxa only group continued with a 5·79‐fold increase from day twelve to day eighteen likely due to the continued tumor proliferation as increased tumor burden and ascites development was observed. While the majority of Glc‐1,6‐BP presence is due to tumor metabolism, we suspect that this metabolite was detectable in the ascites as the high cell turnover and hypoxia from rapid growth resulted in a level of tumor cell death, releasing the metabolite into the surrounding fluid.

5‐HIAA is a metabolite from the breakdown of L‐tryptophan (Trp). Presence of tumors can upregulate indoleamine 2,3‐dioxygenase (IDO), which preferentially degrades Trp into Kyn, a known immunosuppressive metabolite. With lower levels of IDO from lower tumor burden, we would expect higher levels of 5‐HIAA compared to Kyn in Oxa+DSTAP‐R848 treated mice. In the Oxa+DSTAP‐R848 treated group, its levels went through a 3·67‐fold decrease from day ten to day twelve, followed by a 3·38‐fold increase from day twelve to day eighteen. For the Oxa only treatment group, 5‐HIAA was decreased over doses with a 3·03‐fold decrease from day ten to day twelve and then another 2·43‐fold decrease from day twelve to day eighteen (Figure 4C). On the other hand, Kyn increased in concentration over time in the Oxa only group, and decreased in the Oxa+DSTAP‐R848 treated group, suggesting Trp metabolism could be shunted to the Kyn pathway when tumor burden increases (Figure S6). When metabolic profiles were visualized as glycolysis and Krebs cycle heatmaps, the general trend showed an increase in glycolytic metabolites in the Oxa only group over time, whereas there was an overall decrease in the Oxa+DSTAP‐R848 treated group. Oxa+DSTAP‐R848 treated groups appeared to have higher levels of the Krebs cycle metabolites compared to the Oxa only control (Figure S7). Taken together, these data suggest that throughout the treatment regimen, DSTAP‐R848 treatment both reduced tumor burden and drove the immune microenvironment of the peritoneal cavity to a less immunosuppressive state compared to the Oxa only control.

### 3.5 Immunosuppressive and Glycolytic Metabolites Were More Prevalent in Oxa Only Treated Groups

**FIGURE 5:**
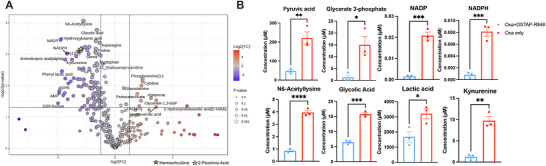
Differences in metabolic differences after dose six between DSTAP‐R848 treated and control groups. (A) Volcano plot of Oxa+DSTAP‐R848 vs. Oxa only groups. Significant metabolites are labelled. (B) Comparison of glycolytic and immunosuppressive metabolites at the day eighteen time point. Data are presented as mean ± standard error mean (SEM). Statistics were performed using an unpaired *t*‐test (^****^, *p* < 0·0001, ^***^, *p* < 0·001, ^**^, *p* < 0·01, ^*^, *p* < 0·05).

As metabolites detected in the peritoneal fluid after day eighteen were the most different between the Oxa only control and Oxa+DSTAP‐R848 treated groups, further analysis was done at this time point to identify potential metabolic pathways that could be up or downregulated. In a volcano plot comparing the metabolites from Oxa+DSTAP‐R848 treated mice to Oxa only control mice, 92 metabolites were significantly lower in abundance while 10 were in higher abundance (Figure 5A). The top hits that were significantly higher when comparing Oxa+DSTAP‐R848 treated groups to Oxa only were phosphoserine (3·76‐fold higher), cytidine (3·74‐fold higher), ethanolamine (3·53‐fold higher) and protocatechuate or protocatechuic acid (2·90‐fold higher).

Some of the top hits of metabolites that were significantly more abundant from the Oxa only control was NADP (13·86‐fold higher), NADPH (10·19‐fold higher), homocitrulline (5·22‐fold higher), and 2‐picolinic acid (4·84‐fold higher). The higher levels of NADP/NADPH were likely due to the general increase in metabolic activity from uncontrolled cancer growth and increased tumor burden [18]. Cellular stress of the TIME can induce post‐translational modification in proteins with the conversion of lysine residues to homocitrulline [19]. An increased presence of myeloperoxidase‐producing MDSCs as found in Figure 3C, combined with hydrogen peroxide from tumors can result in homocitrullination, thus driving the increased levels of homocitrulline in the TIME [20]. Two‐picolinic acid is a metabolite of the kynurenine pathway, and its increase could be related to the IDO enzyme expressed by the tumor, signaling increased immunosuppression [21, 22].

To visualize the increase in glycolysis and general energy consumption from the heavy tumor burden in the Oxa only treated group, several metabolites were chosen in the top row of Figure 5B. Comparing the Oxa only control to the Oxa+DSTAP‐R848 treated group, glycerate 3‐phosphate, an intermediate product of glycolysis was 12·21‐fold higher, pyruvic acid, an end product of glycolysis was 4·66‐fold higher. In the bottom row of Figure 5B, several known or implicated to be immunosuppressive metabolites were visualized. Lactic acid and kynurenine were all higher in abundance in the Oxa only control group thus likely contributing to the immunosuppression that was observed in Figures 2 and 3C. Two hits that were highly significantly different between groups were N6‐acetyllysine (4·62‐fold higher in Oxa only control) and glycolic acid (2·42‐fold higher in the Oxa only control). Neither of the two metabolites have been directly implicated to be immunosuppressive or contributing to cancer growth, so further studies would be needed to identify their role in the tumor environment. Taken together, these data suggest that peritoneal environment of OXA+DSTAP‐R848‐treated mice are the most metabolically different after day eighteen, with a lower abundance of immunosuppressive metabolites compared to the Oxa only control.

## 4 Discussion

In this study, we have developed a cationic DSTAP‐liposome to retain R848 in the peritoneal cavity and found it was synergistic with Oxa resulting in tumor free survival in our preclinical models with the development of durable immune memory upon rechallenge. By profiling the TIME throughout treatment, we were able to identify immune and metabolic fluctuations that occurred alongside a positive response to our immunotherapy.

Incorporation of cationic lipid DSTAP into our liposome resulted in a positive zeta potential, allowing for increased interaction with negatively charged cellular membranes. Its size between 100–200 nm also encouraged uptake, thus resulting in its retention in the peritoneal cavity. By loading R848 into the core of the liposome for delivery, we overcame its typical insolubility in biocompatible solvents [23], as well as localized its inflammatory effects, decreasing the chances of undesirable adverse events [24, 25]. Previous studies have shown that DSTAP‐R848 was primarily taken up by dendritic cells and macrophages in the peritoneal cavity, and this uptake has resulted in an increase in IFN‐α levels in the peritoneal fluid while plasma levels remained low [17].

We combined our therapeutic with Oxa to take advantage of ICD, denoted by the release of CRT, HMGB1 and ATP upon cancer cell death [26]. Two models of PM were used, one with ovarian cancer cells (ID8) and one with colorectal cancer cells (CT26). Both diseases are commonly found to have metastasized to the peritoneal cavity [27, 28]. However, ID8 is known to be more immunosuppressive and have a lower mutational burden, making its treatment with immunotherapies challenging [29, 30]. Additionally, ID8 was less sensitive to Oxa at lower concentrations compared to CT26 in Figure 1D–F, which was more relevant to the achievable in vivo concentrations. Our data resulting in a 30% response rate is encouraging; however, further identification of the mechanism associated with resistance against this therapy will be required to develop cure‐rate improving strategies. Additionally, while the wild‐type ID8 model is commonly used to assess nanoparticle‐based immunotherapies, the ID8*/p53−/−/BRCA1−/−* model has been shown to have the highest overall correlation to patients. This model has also been characterized by a higher MHC‐I expression, so future studies with this model could show different responses with more similarity to human disease [31]. As recurrence is extremely common in ovarian cancers, the ability to resist recurrence is attractive, suggesting further studies could be important to understand the factors behind this vaccination effect. Combination with anti‐PD‐1 appeared to have an antagonizing effect in this model, with anti‐PD‐1 alone also failing to achieve superior results compared to treatment with Oxa only. We had selected anti‐PD‐1 as the currently approved immunotherapy for assessment of additive effects as CD8+ T cells are typically responsible for the anti‐tumor response in both the CT26 and ID8 model [32, 33]. While antagonism has not been observed previously, we suspect it could be due to the therapies inducing pro‐tumoral inflammation and promoting tumorigenesis [34]. Additionally, survival was used as a readout to assess efficacy for improved clinical impact however peritoneal cancer index score is another potential assessment method that is used to characterize disease progression. Future experiments could incorporate this methodology for a more comprehensive understanding of therapeutic efficacy of DSTAP‐R848. Overall, from our data, the ID8 model could respond to immunotherapy but it appears further optimization must be done to ensure adequate efficacy.

Splenocytes were found to be polarized when exposed to peritoneal fluid from mice treated with either Oxa only or Oxa+DSTAP‐R848, with significant increases in immunosuppressive cells in the Oxa only exposed groups. Our metabolic screen showed metabolites like Kyn, lactic acid, and ornithine present in the fluid (Figure 4A). Literature shows evidence of Kyn influencing immunosuppression through the promotion of the Treg‐macrophages suppressive axis in the TME [35], which could contribute to the increase in M2 macrophages and Tregs seen in the splenocytes in Figure 2. Lactic acid has also been shown to polarize macrophages from an M1 phenotype to an M2 phenotype [36]. Additionally, lactic acid supports Treg function, with in vitro experiments demonstrating improved cell viability, expansion and suppressive function [37], likely also contributing to the increased percentage observed. Ornithine also plays a role in cancer, as a substrate that can be decarboxylated to form polyamines to promote tumor growth [38, 39]. Additionally, ornithine has been shown to inhibit T‐lymphocyte activation, potentially resulting in the decreased percentages of activated CD8+ T cells observed [40]. We hypothesize that these immunosuppressive metabolites likely played a role in the polarization of the splenocytes, but whether these had functional effects alone, or in combination with other metabolites has yet to be explored in our study.

Generally, our flow cytometry data supported our hypothesis that the TIME of the CT26 model was being tuned into a less immunosuppressive state with multiple doses of DSTAP‐R848. Our data showed that DSTAP‐liposomes localized in the peritoneal cavity after intraperitoneal delivery, promoting local release of IFN‐a, a key cytokine that activates the immune system [41]. While the percentage of infiltrating DCs was not significantly increased in treated tumors (Figure S4), there may have been a transient activated population as the percentages of CD8+ T cells increased in treated tumors. Imidazoquinoline TLR agonists have been shown to decrease Treg function by suppressing expression of FOXP3, IL10, and TGF‐β [42]. This was in line with our data demonstrating the depletion of Tregs in our Oxa+DSTAP‐R848 treated groups. The decrease in MDSCs by doses three and six are likely due to R848 inducing the maturation and differentiation of these cells. Previous literature has demonstrated that R848 treatment results in the loss of immunosuppressive function in MDSCs and leads to increased antigen presentation [43]. In line with the increased percentage of M1 macrophages by dose six, this could be in part due to MDSC differentiation. Additionally, the exposure of CRT from Oxa administration could also encourage the phagocytic behaviur of macrophages moving to the tumor site [44]. Otherwise, R848 has also been shown to polarize M2 macrophages to an M1 phenotype [45]. Altogether, this data suggests that DSTAP‐R848 was successfully delivered to cells in the TIME and over time, increased cytotoxic immune activity and decreased the immunosuppression and tumor burden.

Metabolic profiles had the biggest differences after the sixth dose or day eighteen of DSTAP‐R848. Most of our results supported the hypothesis of TIME modulation by Oxa+DSTAP‐R848 when compared to the Oxa only control. Trp metabolism appeared to be important in the immunosuppressive or immune‐active nature of the TIME, as noted by the differences in 5‐HIAA to Kyn between Oxa only and Oxa+DSTAP‐R848 treated groups (Figure 4C and Figure S6). As 5‐HIAA levels remained relatively similar in Oxa+DSTAP‐R848 treated groups, we would expect normal levels of IDO1 activity as opposed to in Oxa only groups where IDO1 likely had higher activity due to increased tumor burden, resulting in the increases in Kyn levels (Figure S6). High IDO1 expression and subsequent enrichment in Kyn levels can result in increases of T regulatory cells as well as low tumor infiltration of CD3+ and CD8+ T cells in patients with colorectal cancer and ovarian cancer [46, 47]. This is reflected in our data in Figure 3C where low levels of CD3+/CD8+/IFN‐γ+ infiltration was observed in the Oxa only group. Maintenance of 5‐HIAA levels without increases in Kyn levels suggest the shifting of a suppressive to immune‐active microenvironment upon treatment of Oxa+DSTAP‐R848.

After day eighteen, metabolites like protocatechuic acid, cytidine, ethanolamine and phosphoserine were also found to be enriched in the Oxa+DSTAP‐R848 treated group. Protocatechuic acid is an anti‐oxidative compound that is found in different fruits, vegetables and seeds [48]. As this metabolite is from an exogenous source, its enrichment could be due to an increased appetite in the healthier mice. Cytidine can be used to make cytidine triphosphate, which can then be combined with ethanolamine in the cytidine diphosphate‐ethanolamine pathway to synthesize phosphatidylethanolamine, which has been implicated to be associated with colorectal cancer development [49, 50]. The increase of these two metabolites is potentially linked to the observed decrease in tumor burden. Phosphoserines are often found in biofluids and arise from the proteolysis of proteins containing phosphoserine residues [51]. However, in this context, it is difficult to deduce the reasoning behind their increased abundance so the cause for increased phosphoserine is currently unknown.

Two metabolites that had not been previously implicated as immunosuppressive or immune activating were top hits that found in the screen on day eighteen. N6‐acetyllysine was the most significant hit and has been associated with increased proliferation of colorectal cancer [52, 53]. However, both lysine acetylation and deacetylation have been implicated in cancer progression as well as immune system function so it is challenging to determine the exact role N6‐acetyllysine plays in this context without further experimentation [54, 55]. Future experiments should validate the role of N6‐acetyllysine in colorectal cancers as it could be a targetable vulnerability, or biomarker of prognosis of this disease [56]. Its role in regulating immune activity has yet to be explored as well. Glycolic acid was the second top hit found in this screen. This metabolite has shown to have tumor inhibiting activity in vitro, but has also been previously found in other screens of human patients with later stage colorectal cancers [57, 58, 59]. Its role in tumor progression has not been well established but its presence in different metabolic screens in human colorectal cancer patients does implicate it to be a potential biomarker of the disease. One of the pathways glycolic acid involves is the glyoxylate shunt, which bacteria use for gluconeogenesis and response to oxidative stress [60]. In late stages of colorectal cancer, gut permeability could be increased leading to changes in the microbiota as well as general leakage of bacteria into the peritoneal space, leading to this increase in metabolic activity [61, 62, 63]. Overall, further experiments to validate the importance of glycolic acid in the colorectal cancer environment are warranted.

Generally, this study is a proof of concept of DSTAP‐R848 to further understand the mechanism behind its efficacy and to identify potential biomarkers for treatment response. The six‐dose administration method through the intraperitoneal route can be challenging in a clinical setting. Therefore, future studies to improve translation of treatment would require the development of a sustained release formulation so a one‐time injection would be enough to achieve the same results.

## 5 Conclusion

In summary, we have developed a cationic liposome that delivered and retained R848 in the peritoneal space of our PC models. When administered with Oxa, a high cure rate in the CT26 model of 80% allowed us to investigate the temporal flux of immune cells and metabolites throughout treatments, showing a shift away from an immunosuppressive TIME with repeated dosing of DSTAP‐R848. Beyond the cure rate, we were able to induce specific anti‐tumor immunity in both the colorectal and ovarian PC model with Oxa+DSTAP‐R848, despite the superior response in the colorectal model compared to the ovarian one. Additionally, the profiling of the TIME remodelling both cellularly and metabolically allowed for the identification of biomarkers that could be useful in monitoring efficacy throughout immunotherapy treatment of PC from colorectal cancers. Altogether, this work introduced a nanoparticle‐based therapeutic option that results in the development of long‐term immune memory through TIME polarization, thus providing a promising strategy for the treatment of PC.

## Author Contributions

Conceptualization, **V.C., S.D.L**.; Methodology, **V.C., P.H.C., X.X.S., and J.H**.; Formal analysis, **V.C., X.X.S., and S.M**.; Investigation, **V.C., P.H.C., X.X.S, J.H., S.M., L.J.A., N.B., and T.D**.; Visualization, **V.C**.; Verification, **V.C., P.H.C**.; Writing – original draft, **V.C**.; Writing – review & editing, **V.C., S.D.L**.; Supervision, **S.D.L, M.B., and J.L**. All authors read and approved the final version of the manuscript.

## Conflicts of Interest

The authors declare no conflicts of interest.

## Supporting information




**Supporting File**: adhm71424‐sup‐0001‐SuppMat.docx.

## Data Availability

The data that support the findings of this study are available from the corresponding author upon reasonable request.
