# Supplementary material for: Temporal Immune and Metabolic Shifts Drive the Anti‐Tumor Efficacy of Resiquimod‐Loaded Nanoparticles in Peritoneal Carcinomatosis
Source: Adv Healthc Mater. 2026 Jul 8;15(30):e71424. doi: 10.1002/adhm.71424 (PMC13474142; doi:10.1002/adhm.71424)
Supplement: Supplementary file 1 [file ADHM-15-0-s001.docx]

100 nm

**Supplemental Figure 1.** DSTAP-R848 visualized at 68000X magnification on TEM.

**Supplemental Figure 2.** Stability of DSTAP-R848 at 37^o^C and in storage at 4^o^C. (A) Size of DSTAP-R848 at 37^o^C in 5% glucose. (B) PDI of DSTAP-R848 at 37^o^C in 5% glucose. (C) Percent of R848 encapsulated in storage at 4^o^C. (D) Size of DSTAP-R848 in storage at 4^o^C. (E) PDI of DSTAP-R848 in storage at 4^o^C.

Oxa only

Oxa+DSTAP-R848 (Low Burden)

Oxa+DSTAP-R848 (High Burden)

**Supplemental Figure 3.** Tumour burden of Oxa only and Oxa+DSTAP-R848 treated mice with groups of DSTAP-R848 treated mice separated by tumour burden.

**Supplemental Figure 4.** Flow graphs of dendritic cells, natural killer cells and CD4+ T cells across different timepoints between Oxa only and Oxa+DSTAP-R848 treated groups with high and low tumour burden. Statistics were performed using a one-way ANOVA with a Tukey’s post hoc test or an unpaired t-test.

**Supplemental Figure 5.** Principle Component Analysis of Oxa only and Oxa+DSTAP-R848 treated mice at day ten, twelve and day eighteen timepoints.

**Supplemental Figure 6.** Kynurenine levels across different timepoints in Oxa only and Oxa+DSTAP-R848 treatments. Statistics are performed using one-way ANOVA and Tukey’s post-hoc test (***, p<0.001, *, p<0.05).

**Supplemental Figure 7.** Glycolysis and Krebs cycle metabolites across Oxa only and Oxa+DSTAP-R848 treated mice at day ten, twelve and day eighteen timepoints.

**Supplemental Figure 8.** Gating strategy for different immune subsets assessed. All samples were gated on live immune single cells and then gated into their respective cell types including CD8+/IFNy+ T cells, CD4+/CD25+/FoxP3+ T cells, M1 and M2 macrophages, MDSCs, dendritic cells and NK cells.
